# Positive Selection for New Disease Mutations in the Human Germline: Evidence from the Heritable Cancer Syndrome Multiple Endocrine Neoplasia Type 2B

**DOI:** 10.1371/journal.pgen.1002420

**Published:** 2012-02-16

**Authors:** Soo-Kyung Choi, Song-Ro Yoon, Peter Calabrese, Norman Arnheim

**Affiliations:** Molecular and Computational Biology Program, University of Southern California, Los Angeles, California, United States of America; University of Wisconsin–Madison, United States of America

## Abstract

Multiple endocrine neoplasia type 2B (MEN2B) is a highly aggressive thyroid cancer syndrome. Since almost all sporadic cases are caused by the same nucleotide substitution in the *RET* proto-oncogene, the calculated disease incidence is 100–200 times greater than would be expected based on the genome average mutation frequency. In order to determine whether this increased incidence is due to an elevated mutation rate at this position (true mutation hot spot) or a selective advantage conferred on mutated spermatogonial stem cells, we studied the spatial distribution of the mutation in 14 human testes. In donors aged 36–68, mutations were clustered with small regions of each testis having mutation frequencies several orders of magnitude greater than the rest of the testis. In donors aged 19–23 mutations were almost non-existent, demonstrating that clusters in middle-aged donors grew during adulthood. Computational analysis showed that germline selection is the only plausible explanation. Testes of men aged 75–80 were heterogeneous with some like middle-aged and others like younger testes. Incorporating data on age-dependent death of spermatogonial stem cells explains the results from all age groups. Germline selection also explains MEN2B's male mutation bias and paternal age effect. Our discovery focuses attention on MEN2B as a model for understanding the genetic and biochemical basis of germline selection. Since RET function in mouse spermatogonial stem cells has been extensively studied, we are able to suggest that the MEN2B mutation provides a selective advantage by altering the PI3K/AKT and SFK signaling pathways. Mutations that are preferred in the germline but reduce the fitness of offspring increase the population's mutational load. Our approach is useful for studying other disease mutations with similar characteristics and could uncover additional germline selection pathways or identify true mutation hot spots.

## Author Summary

Multiple endocrine neoplasia type 2B (MEN2B) is a highly aggressive thyroid cancer syndrome. MEN2B offspring with unaffected parents almost always received a new mutation from the father. Moreover, this mutation is almost always at the same nucleotide in the *RET* proto-oncogene. Thus MEN2B's incidence should equal the average single nucleotide mutation frequency, but the observed incidence is 100–200 times greater. One explanation is that the mutation rate at the causal nucleotide is significantly elevated above the genome average. Another is that human testis stem cells acquiring this mutation have a selective advantage over non-mutated ones and this advantage increases the mutation's frequency in the testis. Computational analysis of our testis dissection and mutation assay data rejects the hot spot but not the selective advantage explanation. Because the normal *RET* gene is known to be critical for mouse testis stem cell function, we now have an important insight into what biochemical pathways are altered by the MEN2B mutation to provide this selective advantage in humans. Germline selection explains the unexpectedly high incidence of MEN2B, why the mutation's origin is almost always in the father, and why the probability a child is born with this disease increases with the father's age.

## Introduction

Multiple endocrine neoplasia (MEN) type 2 is characterized by thyroid cancer, variable penetrance of tumors or hyperplasia in other endocrine organs and mutations in *RET*, the receptor tyrosine kinase proto-oncogene “*rearranged during transfection*” [1], [2]. The disease is transmitted in an autosomal dominant fashion. MEN2 has two subtypes: MEN2A (OMIM 171400) accounts for ∼90–95% of cases including a less penetrant sub-form (familial medullary thyroid carcinoma, FMTC [2], [3]), while MEN2B (OMIM 162300) makes up the remaining ∼5–10%.

MEN2B is characterized by a number of interesting genetic features. (1) Half of all new cases result from sporadic mutations, the vast majority (>95%) of which arise in the male germline [4], [5]. (2) The average age of the males who transmit a new mutation to their children is greater than that of the average age of all fathers (paternal age effect [4]). (3) The overwhelming majority (95%, [6]) of new MEN2B mutations occur at the same nucleotide site (c.2943T>C) resulting in the same amino acid substitution (M918T) [7]–[9]. (4) Given that most new cases are caused by mutations at this one site, the incidence of the disease implies that the c.2943T>C nucleotide substitution frequency is several hundred fold greater than the genome average mutation frequency estimated from evolutionary sequence comparisons [10]–[15] and direct disease incidence data [16], [17] (see Text S1 for the detailed calculation).

One possible explanation for the elevated frequency and paternal age effect is that the c.2943T nucleotide site in *RET* is unusually susceptible to undergoing the T>C transition mutation compared to a T elsewhere in the genome (hot spot model). An alternative possibility (germline selection model) is that the c.2943T>C mutation is not unusually susceptible to mutation but, as a result of the biochemical consequences of the MEN2B amino acid substitution, the mutated self-renewing Ap spermatogonial stem cell (SrAp) is provided with a proliferative advantage. (The designations Ap, and Ad which we discuss later in this manuscript, refer to the cytological staining properties of the pre-meiotic A-pale and A-dark spermatogonia, respectively; reviewed in [18]).

There is considerable evidence that the two common Apert syndrome FGFR2 mutations confer a germline advantage on human SrAp (reviewed in [19], [20]). Apert syndrome also shares many of the same interesting genetic features as MEN2B. Unfortunately, the biochemical role normally played by FGFR2 in mammalian SrAp function is virtually unknown [21]–[23]. One advantage, then, to asking whether a germline selective advantage is responsible for the elevated MEN2B mutation frequency and paternal age effect is that wild type RET's role has been extensively studied in mouse testis and is known to be required for the self-renewal of mouse spermatogonial stem cells (SSC, reviewed in [24], [25]). Our observation that the MEN2B mutation does in fact confer a germline selective advantage allows a unique insight into the molecular pathways involved in positive germline selection in human males.

## Results

### MEN2B testes data

**Figure 1:**
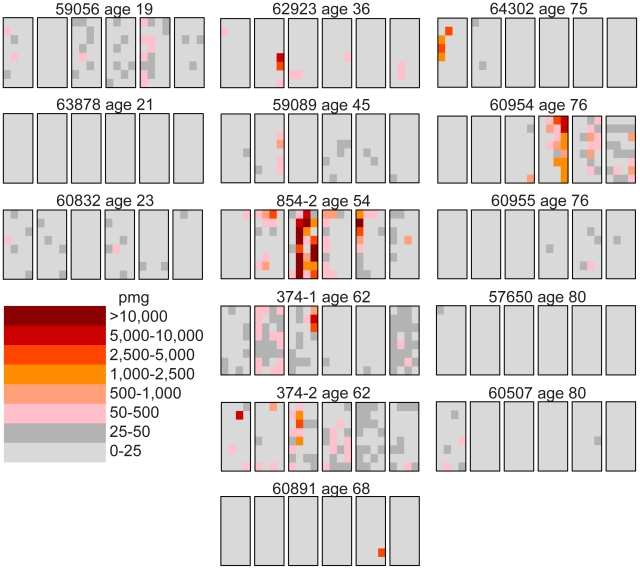
Distribution of the MEN2B mutations in 14 human testes. Each testis is cut into 6 slices, and each slice is further dissected into 32 approximately equal pieces. The mutation frequency per million genomes (pmg) in each piece is represented by the color code in the lower left-hand corner of the figure. Above each testis is the identification number and the age of the donor. The testes are organized by age: the left-hand column is the youngest age group (19 to 23 years), the middle column is the middle-aged group (36 to 68 years), and the right-hand column is the oldest age group (75 to 80 years).

We follow our recently developed approach [26], [27] by measuring the spatial distribution of the MEN2B c.2943T>C mutation in fourteen testes from normal men. We then quantitatively test whether or not these distributions are consistent with the hot spot model that predicts a uniform distribution of SrAp with new mutations or the selection model that predicts these mutant cells will be clustered. Both models assume that the germ cells that undergo mutation are uniformly distributed in the testis (for details supporting this assumption see Text S2). Each testis was cut into 6 slices and each slice into 32 pieces of approximately equal size. The amount of DNA in each piece was quantitated and the frequency of mutant MEN2B molecules was established for each piece using a highly sensitive modification of allele-specific PCR called PAP [28] that gave a false positive rate of 4.7×10^−7^ (based on analysis of ∼2.7×10^8^ control genomes). For each testis piece we estimate the mutation frequency per million genomes (pmg). In Figure 1, this frequency is represented by a heat map with colors ranging from light gray to dark brown. Dataset S1 contains mutation frequency estimates for each testis piece. In Table 1, we use several statistics to summarize this data. For each testis, we consider the average mutation frequency of all the pieces (Av). Many testes have individual pieces with frequencies that are very different from the average. For each testis, we also identify the piece with the maximum mutation frequency (Mx). In order to normalize for the varying average frequencies among different testes, we consider the ratio of Mx to Av in each testis (Mx/Av). In addition, we consider the fraction of testis pieces with mutation frequencies less than 50 pmg (F<50); in Figure 1 these pieces are colored light or dark gray.

**Table 1:** MEN2B mutation frequency summaries from 14 testes.

Testis	Age	Av^a^	Mx^b^	Mx/Av^c^	F<50^d^ (%)
Youngest donors (19–23 years)					
59056	19	15	65	4	95
63878	21	1	13	13	100
60832	23	10	59	6	99
Middle-aged donors (36–68 years)					
62923	36	60	8,348	139	95
59089	45	19	643	34	98
854-2	54	1,188	48,884	41	76
374-1	62	68	5,784	85	90
374-2	62	103	5,843	57	88
60891	68	57	4,964	87	99
Oldest donors (75–80 years)					
64302	75	75	4,372	58	98
60954	76	203	6,673	33	84
60955	76	10	55	6	99
57650	80	1	30	30	100
60507	80	10	56	6	99

a Av testis average mutation frequency per million genomes.

b Mx maximum mutation frequency piece (per million genomes).

c Mx/Av ratio maximum mutation frequency piece to testis average mutation frequency.

d F<50 fraction pieces with mutation frequency less than 50 mutants per million genomes.

### Youngest samples

The youngest age group is made up of three individuals 19, 21, and 23 years of age. For this age group, the Av ranges from 1 to 15 pmg. The Mx ranges from 13 to 65 pmg. Figure 1 shows that all the pieces' mutation frequencies are colored light gray (<25 pmg), dark gray (25 to 50 pmg), or pink (50 to 500 pmg). The few pieces colored pink in this age group are in the low end of the pink range, since the one with the greatest frequency is only 65 pmg. For each testis the F<50 ranges from 95% to 100%.

### Middle-aged samples

Six individuals, aged 36 to 68 years, comprise the middle-aged group. For these testes, the Av ranges from 19 to 1,188 pmg. In contrast to the youngest age group, each testis has a small number of pieces with mutation frequencies that are several orders of magnitude greater than the remaining pieces. The Mx ranges from 643 to 48,884 pmg. These high frequency pieces are more darkly colored in Figure 1, and are often clustered together in the same slice or in adjacent slices. The sample with the lowest Av (#59089) also has the lowest Mx, and the sample with the highest Av (#854-2) also has the highest Mx. The Mx/Av ratio ranges from 34 to 139. The F<50 fraction is still high, ranging from 76% to 99%.

Both the Av and the Mx are greater for the middle-aged group than the youngest age group. However, within the middle-aged group there is no obvious correlation between frequency and age. Indeed the testis with both the lowest Av and Mx is from a 45 year old (#59089), and the testis with both the highest Av and Mx is from a 54 year old (#854-2). So the extreme frequencies come from individuals with ages in the middle of the group, and with ages that are close to each other.

### Oldest samples

The oldest age group containing five individuals aged 75 to 80 years is heterogeneous. Two of the individuals (#64302 and #60954) have frequency values typical of the middle-aged group: the Av are 75 and 203 pmg, the Mx are 4,372 and 6,673 pmg, the Mx/Av are 58 and 33, and the F<50 are 98% and 84%. The remaining three individuals (#60955, #57650, and #60507) have much lower frequency values typical of the youngest age group: the Av are 10 pmg or less, the Mx are 56 pmg or less, and the F<50 are 99% and 100%. The three low frequency old samples will be further discussed later in the Results section. For discussion purposes, we define a testis as having “substantial” mutation clusters if Mx is greater than 500 pmg: this group includes all of the middle-aged samples and the first two from the oldest age group, while excluding all of the youngest samples and the last three from the oldest age group.

### Hot spot model

Previously, we developed a model based on what is known about human germ-line development and maturation to quantitatively test whether the mutation distribution in a testis is consistent with a hot spot model [26], [27]. Here we briefly review the model, apply it to the c.2943T nucleotide site in the *RET* gene, and discuss a new variant to the model. The computer programs to simulate all the models discussed here and elsewhere in the paper can be found in Protocol S1.

The hot spot model has two phases that we call the growth-phase and the adult-phase. The growth-phase models the testis from zygote formation to puberty. During this phase, divisions of the male germ-line cells are symmetric and self-renewing, and the number of such cells increases exponentially. Similar to a Luria and Delbruck “mutation jackpot” in bacteria [29], a mutation arising early in this phase will be shared by more descendent germ-line cells than will later mutations. The primordial germ cells migrate to the site of gonad formation and form the seminiferous cords early in fetal development [30], [31] and since germ cells are expected to remain physically close to their ancestors once the chords are formed, further cell divisions of early mutations can result in mutation clusters. There are approximately 30 growth-phase generations [32].

The germ-cells originating during the growth-phase eventually form the adult SrAp. These cells cycle throughout a man's life providing many opportunities for new mutations. The adult-phase portion of the model considers the testis after puberty. During this phase, the SrAp divide asymmetrically to produce a daughter SrAp (self-renewal) and another daughter cell whose descendants, after a few additional divisions, will produce sperm. In an adult male, the SrAp divide every 16 days [33], and therefore from an individual's age we can estimate the number of adult phase generations that his SrAp cells have experienced. In our model any new mutation in the adult phase can produce only one mutant SrAp self-renewing cell lineage. The model has only one free parameter: the mutation rate per cell division.

For each testis, the data is the mutation frequencies of the 192 testis pieces. In order to test the hot spot model using the maximum likelihood approach one would need to calculate the probability, as a function of the model parameters, of the mutation frequencies for all 192 testes pieces. Unfortunately, none of the models we consider are amenable to such calculations. One could estimate this probability function by counting the number of computer simulations of the model that exactly match all 192 frequencies. However, the probability of exactly matching all 192 frequencies is so low that this approach is not feasible. The goodness-of-fit strategy that we pursue instead is we test whether there are values of the model parameters such that computer simulations of the model can approximately match the three summary statistics Av, Mx/Av, and F<50 simultaneously. These statistics attempt to summarize both the mutation frequency and the clustering observed in the testes. Say, for example, a model predicts a more uniform distribution of frequencies than was observed so that simulations which approximate the observed Av statistic also feature much lower than observed Mx/Av ratios. Since this model fails to capture both the mutation frequency and the clustering observed in the testis, we would reject such a model. Alternatively, suppose another model approximately matches the three summary statistics simultaneously. Since this model reproduces both the mutation frequency and the clustering observed in the testis, we would declare such a model consistent with the data.

Let us consider the example of testis #374-1 from a 62 year old. The observed Av is 68 pmg (Table 1). In simulations, we vary the mutation rate per cell division until we find the value of this model parameter such that the simulated Av best matches the observed Av. We simulate the model using this parameter value until we have one million simulations where the simulated Av is within 5% of the observed Av, and then we compare the other statistics for these simulations (Table 2) to the actual data. For the observed data the Mx/Av ratio is 85, while in 95% of simulations the ratio is between 2.1 and 4.3. Indeed, in one million simulations this ratio is always less than was observed in the data. Similarly the observed Mx is 5,784 pmg, while in 95% of simulations the Mx is between 144 and 288 pmg. Since we only consider those simulations such that the simulated Av is within 5% of the observed Av, the results for the two statistics Mx and Mx/Av closely correspond. Since we find the ratio Mx/Av more intuitive, we will only consider it subsequently. Likewise, for the data the F<50 statistic is 90%, while in 95% of simulations this fraction is between 25% and 35%. In one million simulations this fraction is always less than was observed in the data. Thus we are able to strongly reject the hot spot model with p-value less than 10^−6^. In Table 2, we see the same conclusion holds for the remaining seven testes with substantial mutation clusters. Note that for testis #59089 in 95% of simulations the F<50 statistic is between 99% and 100% because the Av (19 pmg, Table 1) is less than 50 pmg.

**Table 2:** Hot spot model parameter and simulation results for those testes with substantial MEN2B mutation clusters.

		Model parameter	Mx/Av^a^			F<50^b^ (%)		
Testis	Age	Mutation rate per cell division	Data	Simulated 95% range	p-value	Data	Simulated 95% range	p-value
Original hot spot model								
62923	36	1.24×10^−7^	139	1.9–6.1	<10^−6^	95	38–49	<10^−6^
59089	45	2.40×10^−8^	34	2.4–4.9	<10^−6^	98	99–100	1.0
854-2	54	1.38×10^−6^	41	2.0–4.3	<10^−6^	76	0–0	<10^−6^
374-1	62	6.62×10^−8^	85	2.1–4.3	<10^−6^	90	25–35	<10^−6^
374-2	62	1.01×10^−7^	57	1.9–3.9	<10^−6^	88	1–5	<10^−6^
60891	68	5.00×10^−8^	87	1.9–3.7	<10^−6^	99	44–54	<10^−6^
64302	75	5.82×10^−8^	58	2.1–3.9	<10^−6^	98	16–24	<10^−6^
60954	76	1.54×10^−7^	33	1.9–3.9	<10^−6^	84	0–0	<10^−6^
Symmetric variant								
62923	36	1.24×10^−7^	139	3.8–9.7	<10^−6^	95	54–63	<10^−6^
59089	45	2.40×10^−8^	34	9.6–22.8	0.01	98	88–93	<10^−6^
854-2	54	1.38×10^−6^	41	2.5–5.2	<10^−6^	76	0–1	<10^−6^
374-1	62	6.62×10^−8^	85	5.6–12.9	<10^−6^	90	55–70	<10^−6^
374-2	62	1.01×10^−7^	57	5.7–12.3	4×10^−6^	88	52–59	<10^−6^
60891	68	5.00×10^−8^	87	7.3–15.8	<10^−6^	99	71–78	<10^−6^
64302	75	5.82×10^−8^	58	5.6–15.1	9×10^−6^	98	53–72	<10^−6^
60954	76	1.54×10^−7^	33	4.3–8.9	10^−4^	84	35–44	<10^−6^

a Mx/Av ratio maximum mutation frequency piece to testis average mutation frequency.

b F<50 fraction pieces with mutation frequency less than 50 mutants per million genomes.

In the hot spot model, a mutation early in the growth phase could produce a mutation cluster. In order to match the observed Av, however, the inferred value of the mutation rate per cell division model parameter is low enough such that mutations early in the growth phase are rare. Since the SrAp divide every 16 days, in a 62 year old male there have been approximately 500 times more adult phase cell divisions than growth phase divisions [27] and mutations in the adult phase do not produce mutation clusters. Consequently, in simulations of the hot spot model that match the observed Av the distribution of mutations is more uniform than observed. Furthermore, even if one does not agree with the modeling details, the youngest age group has markedly lower Av and Mx statistics than the middle-aged group (Table 1). Therefore, the increase in the mutation frequencies and the growth of the mutation clusters occurs in the adult, not during development.

Finally, we previously examined the distribution of a likely neutral mutation in testis samples using the same approach [27]. We assayed a C to G mutation in the intron of the CAV1 gene on chromosome 7. This presumably neutral mutation was studied in testes 374-1 and 374-2 (62 years of age) and involved the same DNA samples we used for the MEN2B analysis. The summary statistics are identical for both testes (Av = 3, Mx = 20, Mx/Av = 6.67 and F<50 = 100%) and similar to the MEN2B data from much younger donors. Simulations showed that the relatively uniform distribution of mutations was consistent with the hot spot model.

### Symmetric hot spot model variant

Based on work in the mouse [34] and human [35], we also consider a variant to the hot spot model where the SrAp in the adult phase, independent of whether or not they have acquired the disease mutation, may divide symmetrically. As in the original model, each SrAp in the adult phase divides every 16 days, but now there are three possible types of divisions (the probabilities of these types sum to one). This variant introduces a second model parameter q. With probability 1-2q, the SrAp cells divide asymmetrically as in the original hot spot model. However, now with probability q, the SrAp cells divide symmetrically producing two SrAp cells: both daughter SrAp cells share any accumulated mutations and since these cells remain physically near each other, multiple symmetric divisions would produce a mutation cluster. Also with probability q, in order to keep the number of SrAp cells approximately constant [36], [37], an SrAp cell can produce two differentiated daughter cells (B spermatogonia) that both go on to make sperm thus eliminating one SrAp cell lineage.

For a given mutation rate per cell division the Mx/Av and F<50 statistics increase with the value of the symmetric parameter q, therefore to make the test as conservative as possible we only consider the case where q equals the maximum possible value 0.5 (so one-half of the divisions produce two SrAp cells and one-half produce two B spermatogonia). As in the test of the original hot spot model, we simulate the model with the mutation rate per cell division that best matches the observed Av until we have one million simulations with Av within 5% of the observed Av. We again consider sample #374-1. For the data the Mx/Av ratio is 87, while in 95% of simulations this ratio is between 5.6 and 12.9. For the data F<50 is 90%, while in 95% of simulations this fraction is between 55% and 70%. The symmetric variant to the hot spot model increases these statistics greater than the level achieved by the original hot spot model, but not as high as is observed for the data. Since in one million simulations both the Mx/Av ratio and the F<50 fraction were always less than was observed in the data, this variant is also strongly rejected with p-value less than 10^−6^. As shown in Table 2, the same conclusion holds for the other testes with substantial mutation clusters.

### Selection model

Previously, in order to explain the mutation clustering for the Apert syndrome mutations, we had proposed a role for selection [26], [27]. The selection model is based on the original hot spot model, and adds a selection parameter p: at each adult phase generation, a mutated SrAp divides symmetrically with probability p and asymmetrically with probability 1-p (after a symmetric division, each daughter SrAp reverts to asymmetric divisions until the next rare symmetric division). A similar model was proposed by Crow [38]. Unlike the symmetric hot spot model considered above, non-mutated SrAp cells in the adult phase can only divide asymmetrically. Since the SrAp daughter of an SrAp cell is expected to remain near its progenitor, these rare symmetric divisions can cause mutation clusters to form and grow locally over time. The motivation for the selection model is that in model organisms it has been shown that stem cells can switch from asymmetric to symmetric divisions and back again, and that such behavior can depend on factors intrinsic and extrinsic to the stem cells [39], [40].

Consider again sample #374-1 for the MEN2B mutation. The selection model has two free parameters: the mutation rate per cell division and the selection parameter p. With these two free parameters, we can now try to match both the Av and the Mx. As before, we only consider those simulations such that the simulated Av is within 5% of the observed Av. For the data the Mx/Av ratio is 85, and in 95% of simulations this ratio is between 26 and 92. For the data the F<50 fraction is 90%, and in 95% of simulations this fraction is between 86% and 93%. Therefore the selection model is consistent with the data for this testis. The inferred selection parameter p is 0.0084, so if the mutated SrAp cells divide symmetrically approximately 1% of the time, this is sufficient to form mutation clusters similar to what is observed in the testes. Moreover, if we now take the inferred mutation rate per cell division (4.4×10^−11^) and set the selection parameter p equal to zero, then simulations of the model produce mutation frequencies similar to the already established genome averages [10]–[17], implying that the mutation rate per cell division is not elevated at this nucleotide [26], [27].

The selection model can explain the paternal age effect since the mutation clusters will grow as the man ages and the male mutation bias since this growth is only in the male germline. The selection model can also explain the elevated mutation frequencies and the clustering observed for all the other testes with substantial mutation clusters (results not shown). However, this model predicts that the samples in the oldest age group will have the highest mutation frequencies and the most intense mutation clusters, and thus cannot explain the low mutation frequencies and lack of mutation clusters observed in three of the testes from this age group (see next heading).

We have also considered a “combined” model which merges the symmetric variant to the hot spot model with the original selection model: all SrAp randomly divide asymmetrically, divide symmetrically or divide to produce two differentiated daughter cells, but the mutant SrAp are more likely than the wild type SrAp to divide symmetrically. However, we did not pursue this combined model further since it introduces an additional model parameter without improving the fit of the selection model.

### Heterogeneous oldest age group

**Figure 2:**
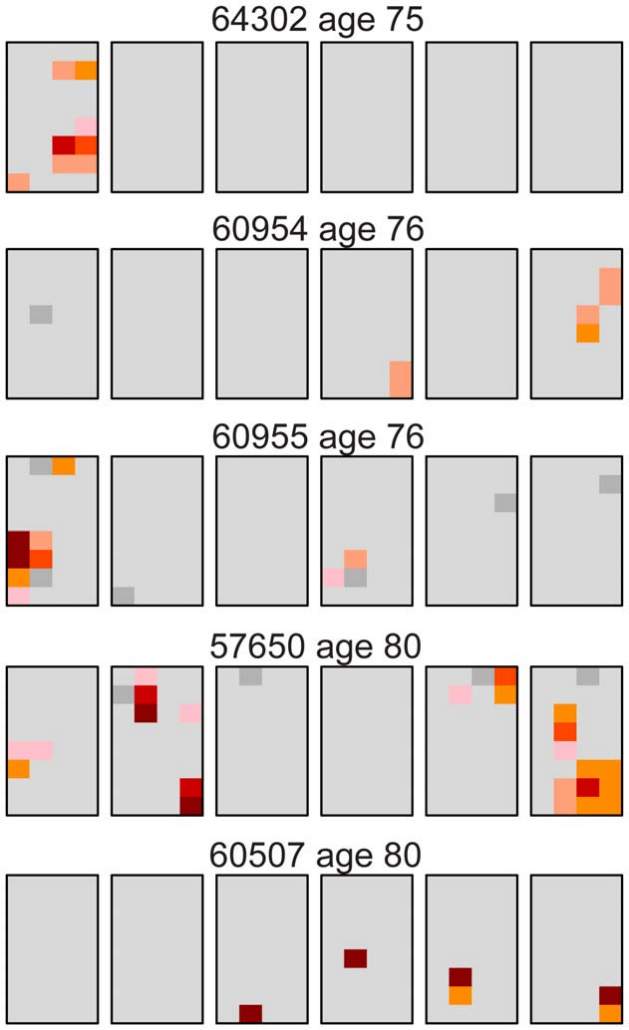
Distribution of the Apert syndrome c.755C>G mutation for the oldest age group (75–80 years). These are the same testes as the oldest age group shown in Figure 1. For the Apert mutation, unlike the MEN2B mutation in Figure 1, all of the older testes have substantial mutation clusters. The color code is the same as in Figure 1.

Our results for the oldest individuals were surprising in that three (#60955, #57650, and #60507) of the five samples had unexpectedly low levels of the c.2943T>C MEN2B mutation similar to young testes (Table 1). One trivial explanation for such low levels of mutation was germ cell degradation in these three older samples and that this data should be discarded. To examine this question we looked at the distribution of a different mutation. We used the version of our assay originally designed for the Apert syndrome c.755C>G mutation [26], [27] on the same 14 testes we studied for the MEN2B mutation (plus one 21 year old sample, #63205, which we had not studied for MEN2B). Figure S1 shows the Apert syndrome mutation distribution for all the testes and Dataset S2 contains the mutation frequency estimates for every piece. Table 3 summarizes the mutation frequency statistics for each testis and Figure 2 shows the mutation distribution for the five testes in the oldest age group. The results showed substantial Apert mutation clusters in all five older testes including those with the fewest MEN2B mutations. Therefore general germ cell degradation in the three testes cannot explain the heterogeneity in the MEN2B data. Another observation, which will play a part in the subsequent modeling, is that for the middle-aged group of testes the median Av is ∼4-fold higher and the median Mx is ∼3-fold higher for the Apert mutation compared to the MEN2B mutation (see Table 1 and Table 3).

**Table 3:** Apert syndrome c.755C>G mutation frequency summaries from 15 testes.

Testis	Age	Av^a^	Mx^b^	Mx/Av^c^	F<50^d^ (%)
Youngest donors (19–23 years)					
59056^e^	19	16	4,000	250	99
63205^f^	21	1	8	8	100
63878	21	2	12	6	100
60832^e^	23	1	9	9	100
Middle-aged donors (36–68 years)					
62923	36	3	2,936	979	99
59089^e^	45	160	8,000	50	93
854-2^e^	54	680	47,000	69	93
374-1^e^	62	380	27,000	71	88
374-2^e^	62	67	7,000	104	97
60891	68	861	45,075	52	95
Oldest donors (75–80 years)					
64302	75	166	6,847	41	96
60954	76	25	2,045	82	97
60955	76	132	18,327	139	95
57650	80	446	36,652	82	88
60507	80	621	30,418	49	97

a Av testis average mutation frequency per million genomes.

b Mx maximum mutation frequency piece (per million genomes).

c Mx/Av ratio maximum mutation frequency piece to testis average mutation frequency.

d F<50 fraction pieces with mutation frequency less than 50 mutants per million genomes.

e previously published [26].

f testis not studied for MEN2B.

### Selection model incorporating cell death

To try and explain the MEN2B data on the oldest age group, we concluded that the only acceptable model modification was to incorporate age-dependent cell death. Researchers have shown that the number of SrAp cells decreases as men grow old [36]: from the ages of 31–40 to 61–70 there is a slight decrease from 162 cells per mm^2^ of seminiferous tubule cross section to 120 per mm^2^, but from the ages of 61–70 to ages 81–90 there is a much more rapid decrease from 120 mm^2^ to 57 per mm^2^. There is a similar pattern of decrease for the A-dark spermatogonia (Ad). Believed to act as “reserve” stem cells, the Ad remain quiescent until the number of SrAp cells is sufficiently diminished to activate the Ad to replace the SrAp [41]. Since the Ad have not been cycling as frequently as the SrAp until this point, they are less likely to have acquired any mutations, and thus the pool of SrAp cells is replenished with a fresh supply of primarily non-mutated cells. We have incorporated cell death into the selection model by assuming that all SrAp, whether or not they are mutated, die at the same rate. The details of this new model can be found in Text S3.

The selection model incorporating cell death can explain all of the testes data for both MEN2B and Apert syndrome. For those testes with substantial MEN2B mutation clusters, as before, we varied the mutation rate per cell division and the selection parameter to try to match both the Av and the Mx, and we only considered those simulations such that the simulated Av was within 5% of the observed Av. Table 4 shows that this model is consistent with these testes. For those testes without substantial mutation clusters, we did not fit each testis separately (many low values of the model parameters would suffice) but rather for a given age and set of parameter values we simulated the model many times to see how often the simulations were typical of a young donor and how often they were typical of a middle-aged donor (see Text S3 for details). For MEN2B, we found that when we set the selection parameter at the low end of the range in Table 4 then most simulations of an older individual were typical of a young donor. However, when we increased the selection parameter to the median value in Table 4 then most simulations of an older individual were typical of a middle-aged donor. Thus a relatively slight variation in the selection parameter between individuals can explain the heterogeneity in the older donors for MEN2B. As for the Apert syndrome mutation, the Av and Mx values in Tables 1 and 3 are greater for the Apert mutation than the MEN2B mutation, leading to slightly higher inferred values for the selection parameter for the Apert mutation (see Table S1). When we increased the selection parameter to the median value for the Apert mutation in Table S1 then almost all of the simulations of an older individual were typical of a middle-aged donor in agreement with Table 3. The slight increase in the value of the selection parameter for Apert syndrome compared to MEN2B can explain the difference in the oldest age groups for these two mutations. Finally for MEN2B and the youngest donors, even using the greatest values of both the mutation rate per cell division and the selection parameter from Table 4, almost all simulations of a 23 year old are typical of a young donor in agreement with Table 1. For these parameter values, the probability of a substantial mutation cluster developing in a 23 year old is very small due to the relatively low number of adult phase generations.

**Table 4:** Selection model incorporating cell death model parameters and simulation results for those testes with substantial MEN2B mutation clusters.

		Model parameters		Mx/Av^a^		F<50^b^ (%)	
Testis	Age	Mutation rate per cell division	Selection parameter	Data	Simulated 95% range	Data	Simulated 95% range
62923	36	3.9×10^−11^	0.020	139	41–149	95	92–97
59089	45	7.0×10^−11^	0.012	34	19–61	98	91–98
854-2	54	9.1×10^−11^	0.013	41	21–79	76	69–80
374-1	62	4.4×10^−11^	0.0084	85	26–92	90	86–93
374-2	62	4.9×10^−11^	0.0088	57	28–91	88	84–92
60891	68	1.5×10^−11^	0.010	87	51–161	99	93–99
64302	75	2.0×10^−11^	0.012	58	43–160	98	92–98
60954	76	1.2×10^−10^	0.011	33	18–61	84	73–84

a Mx/Av ratio maximum mutation frequency piece to testis average mutation frequency.

b F<50 fraction pieces with mutation frequency less than 50 mutants per million genomes.

## Discussion

A highly sensitive DNA amplification method to detect the MEN2B c.2943T>C mutation was combined with a dissection protocol allowing construction of a three dimensional representation of the anatomical distribution of the mutations in the testis. For all the middle-aged testes we studied (ages 36 to 68), the MEN2B mutation frequency was greater than expected based on the genome average mutation frequency. Further, the mutations were clustered: each of these testes had a small number of pieces with mutation frequencies several orders of magnitude greater than the remaining pieces.

Moreover, for the younger testes (aged 18 to 23) there were almost no mutations suggesting that the clusters in the middle-aged donors are not due to mutations arising early in development but grew during adulthood. The hot spot model where SrAp divisions are always asymmetric was rejected (p-value<10^−6^), because it could not reproduce these clusters. A new symmetric variant to the hot spot model in which all SrAp, regardless of whether or not they are mutated, sometimes randomly divide asymmetrically, divide symmetrically to make two SrAp cells or divide to produce two differentiated daughter cells, was also rejected.

The explanation of our data requires some form of germline selection so that mutant SrAp are able to increase in frequency locally and grow clusters. The form of selection we considered is that wild type SrAp always divide asymmetrically, while mutant SrAp generally divide asymmetrically but, critically, occasionally divide symmetrically to produce two SrAp. Our data on testes with substantial mutation clusters fits this model well. The inferred mutation rate per cell division for the selection model is not elevated above that inferred from the genome average mutation frequency using evolutionary sequence comparisons [10]–[15] and direct disease incidence data [16], [17]. Germline selection can also explain the paternal age effect and the male mutation bias, two characteristics MEN2B shares with some other diseases with elevated frequencies (reviewed in [19], [20], [38], [42], [43]).

These results are similar to those we found previously when studying the mutations that cause Apert syndrome [26], [27]. One difference between the MEN2B and Apert mutations is that in older donors (ages 75 to 80) the MEN2B mutation frequency was heterogeneous: some of the older testes were like the middle-aged testes while other older testes were like the younger testes. We were able to explain this observation by incorporating data [37] on age-dependent spermatogonial cell death in the selection model and allowing a slight variation in the value of the selection parameter between individuals. For the Apert mutation, all of the older testes were like the middle-aged testes. A greater value for the selection parameter for the Apert mutation compared to the MEN2B mutation (supported by both a greater median value for the average mutation frequency (Av) and a higher median value for the maximum piece mutation frequency (Mx) for the Apert mutation) can explain this difference.

### MEN2B testicular phenotypes

The MEN2B mutation causes aggressive malignant tumors in the thyroid gland during early childhood and later-appearing tumors in the adrenal glands (pheochromocytoma) as well as other kinds of abnormal growths [2], [44] but testis cancer has not been reported as a feature of men with the disease ([2] updated 5/4/2010). A study of the most common type of human testis cancers (seminomas) and rare spermatocytic seminomas both failed to find tumors carrying the MEN2B mutation [45], [46]. Using a mouse model of MEN2B [47] testicular cancer was not observed in either homozygotes (*Ret^MEN2B/MEN2B^*) or heterozygotes *Ret^MEN2B/+^*. Men with MEN2B disease (carrying the mutation in all their cells) can father children with MEN2B [4], and *Ret^MEN2B/MEN2B^* and *Ret^MEN2B/+^* mice also show normal sperm production [47]. These results indicate that the functional properties of the MEN2B protein are consistent with normal spermatogenesis and spermiogenesis but do not contribute to germ cell tumor formation. On the other hand hyperactivation of RET in *Ret^+/+^* mice by overexpressing glial derived neurotrophic factor (GDNF), the major ligand of RET in the testis, results in germ cell tumors that are seminoma-like and led to disruption of normal spermatogenesis [48]. We conclude that the activating effect of the MEN2B mutation on RET function in SrAp cells is minimal compared to RET function in the mouse germline when continually activated by GDNF. A sufficient selective advantage relative to wild type SrAp is necessary to allow cluster formation yet the achievement of this advantage by MEN2B SrAp cells still allows the production of the differentiating daughter spermatogonia needed for normal sperm production and transmission to the next generation.

### How RET functions in self-renewing germ cells

To understand how MEN2B RET alters the normal function of SrAp cells we must consider RET's normal biochemical properties (reviewed in [44], [49], [50]). RET is a receptor tyrosine kinase activated after forming a complex with both GDNF and a member of the GDNF-family α co-receptors anchored to the cell surface (GFRα1). Complex formation results in RET dimerization and induction of RET's tyrosine kinase activity resulting in trans-autophosphorylation of critical tyrosines in each RET monomer's intracellular domain. Interactions between these phosphorylated tyrosine docking sites and adapter or signaling proteins initiates a variety of downstream signaling pathways.

Wild type RET functions in a wide variety of cells and tissues including the central and peripheral nervous system, during the development of the kidney and in a variety of other organs (including testis). Whether cell proliferation, cell survival, differentiation or a myriad of other cell processes are stimulated or inhibited by RET activation depends on which RET protein isoforms are expressed, the specific cell types involved, their developmental stage and the expression patterns of many additional proteins that function in downstream signaling pathways including Ras/MAPK, SFK and PI3K/AKT among others (reviewed in [44], [49], [50]). Since published work on RET's direct role in the adult human testis is extremely limited we look to studies of the mouse's RET protein for help in understanding how a new MEN2B mutation in a wild type testis might lead to a germline selective advantage of the newly mutated cell in humans. RET signaling is critical for the continuing self-renewal of spermatogonial stem cells in the mouse (SSC) and thus spermatogenesis (reviewed in [23], [24]). Self-renewal requires balancing the number of SSC divisions that lead to more SSCs against SSC divisions that produce precursors of differentiating spermatogonia so that both the number of stem cells and the amount of sperm production is sustained throughout life [23], [25], [51]–[53]. We suggest that pathways involved in maintaining this balance are subtly modified by the M918T mutation.

Using mouse knockout and overexpression models, experiments on *Gdnf*, *Gfrα*1 or *Ret* have shown that all three genes are critical for SSC self-renewal. Other experiments using testis cell cultures grown in serum-free chemically defined media showed that GDNF alone could promote SSC self-renewal for long periods of time [54] implicating RET as being critical for this process. Transgenic mice carrying a phenylalanine mutation (Y1062F) at one of RET's critical tyrosines [55] lose all germ cells within three weeks suggesting that Y1062 function influences this process.

Studies *in vitro* using mouse undifferentiated spermatogonial cell cultures grown in serum-free media showed that the PI3K (phosphatidylinositol-3 kinase)/AKT and SFK (SRC family kinases) pathways play an important role in SSC self-renewal [23], [25], [51]. It has also been proposed that RAS activation is involved in SSC self-renewal [53].

### The effect of the human MEN2B mutation on RET function

The biochemical consequences of the M918T mutation on RET function have been studied extensively in a variety of tissues and cell types but not the germline (reviewed in [44], [49], [50], [56]). The M918T protein can be activated as a monomer even before being inserted into the cell membrane unlike wild type RET, which normally requires dimerization through ligand binding at the cell surface. The mutant protein also alters its own pattern of tyrosine autophosphorylation in the intracellular domain of RET. This can result in weakened signaling for some downstream pathways and/or activation of pathways not normally signaled by wild type RET. Notably, the M918T mutant protein also shows constitutively high levels of tyrosine phosphorylation especially at Y1062, the docking site that influences SSC self-renewal in mice. Proteins that normally bind to Y1062 in wild type RET might be expected to signal the PI3K/AKT, and/or RAS/MAPK downstream pathways at unusually high levels in the testis. Constitutive phosphorylation of the RET binding site (Y981) for SRC (a SFK member) would also be expected to affect SRC signaling.

Based on the role of RET in mouse SSC self-renewal we propose that the M918T mutation modifies the signal transduced from GDNF activated RET downstream through the SFK, PI3K/AKT and possibly RAS pathways leading to the production of mutation clusters. In the human context self-renewal can be achieved by balancing asymmetric and the two types of symmetric SrAp cell divisions as described by our models. The details of how the SFK, PI3K/AKT and possibly RAS pathways enable normal SrAp self-renewal in the germline and how the MEN2B mutation alters these processes to confer a selective advantage to mutated SrAp cells are yet to be determined.

### Similarities between the MEN2B and Apert syndrome mutation processes

Apert syndrome (OMIM 101200) is characterized by premature closing of the sutures between the bones of the skull (craniosynostosis) due to gain of function mutations in the receptor tyrosine kinase fibroblast growth factor receptor 2 gene (*FGFR2*), one of four such receptors (*FGFR*1-4). The disease manifestations of MEN2B and Apert syndrome are very different yet, like MEN2B mutations, new Apert syndrome germline mutations also arise at an unexpectedly high frequency (100–1,000 times that expected) at a limited number of nucleotide sites (c.755C>G or c.758C>G), virtually always occur in the male parent and exhibit a paternal age effect (reviewed in [19], [38], [42], [43], [57]). Both Apert syndrome mutations are distributed in testes as clusters rather than uniformly ([26], [27] and this paper) and, together with other results [22], [38], [58]–[61], support the idea that the unexpectedly high frequency of the two Apert nucleotide substitution mutations and paternal age effect results from a selective advantage acquired by mutated SrAp cells over wild type cells.

### Relevant similarities between RET and FGFR2 cell signaling

Normal FGFR2 activation follows ligand binding to a subset of the 18 human fibroblast growth factors [62], dimerization and transphosphorylation. FGFR2 downstream signaling pathways can influence cell proliferation, cell survival, differentiation and a myriad of other cell functions in many different cell and tissue types (reviewed in [63]–[67]).

Unfortunately, compared to RET, virtually nothing is known about the downstream signaling pathways stimulated by activated FGFR2 in mouse undifferentiated spermatogonial cultures or other germ cells [21]–[23] although the addition of one of the fibroblast growth factors (basic bFGF/FGF2), also a ligand of FGFR2, is required for the self-renewal of mouse SSC in cell culture (reviewed in [23]).

The two Apert syndrome mutations increase FGFR2's FGF ligand binding affinity and alter ligand specificity. Consequently, FGFR2 has an unusually long residence in the cell membrane during which time interactions with other proteins could produce abnormally persistent downstream activation signals [63]–[66], [68]–[72]. Whether mouse or human SSC self-renewal also involves FGFR2 signaling through the PI3K/AKT and SFK pathways or some other pathway is not known although activated FGFR2 can signal through the Ras/MAPK and PI3K/AKT (and possibly SFK [73]) pathways in non-germline cells and tissues.

More information about the normal function of RET and FGFR2 in spermatogonia are needed to provide an explanation for how both these disease mutations might perturb the signaling landscape to elicit a positive germline selective advantage. Our discovery that MEN2B seems to provide a spermatogonial selective advantage will make it possible to leverage the information on the function of RET with regard to what may be learned about FGFR2 and *vice versa* and to find out the similarities and differences between the pathways that lead to a germline selective advantage in each case.

### The significance of germline selection

In a theoretical analysis twenty years ago Hastings [74], [75] studied the consequences of germline selection on the mutational load of a population. The focus of Hastings' work was primarily concerned with very rare recessive alleles already existing in the population but primarily found only in heterozygotes. Hastings suggested that germline genetic events leading to loss of heterozygosity (e.g. gene conversion, mitotic crossing over) in an individual heterozygous for the rare allele could produce premeiotic germ cells homozygous for this allele. If homozygosity for this allele was selectively disadvantageous in both the germline and at the level of an individual the potential mutational load of this allele on the population would be significantly reduced. Hastings also suggested that rare recessive but selectively advantageous alleles could increase in frequency in the population by the same reasoning. Finally, he recognized the possibility that some mutations may have a germline selective advantage but also a selective disadvantage for individuals in the population. This would create what he called a “mitotic drive” system and increase the mutational load of the population. The MEN2B and Apert mutations seem to be realizations of this idea. In both examples the mutation incidence and the magnitude of the paternal age effect are markedly greater than would be expected simply by an increase in the mutation rate per cell division.

There are additional candidate *de novo* disease mutations at other loci that might also provide a germline selective advantage (reviewed in [19], [20], [38], [42], [43]) to spermatogonia. Of course the ability of any such mutation to increase the human mutational load depends upon not significantly interfering with the state of differentiation of the stem cell so as to permit mutant sperm formation and transmission to the next generation. As we learn more about the germline signaling pathways involved and the effect of specific mutations on those pathways it may be possible to identify other mutations that might either provide a selective advantage and be transmitted to the next generation or those that will not be transmitted to the next generation in spite of their germline selective advantage because they interrupt a fundamental aspect of spermatogonial differentiation. The testis dissection method can be useful in studying these questions in animals.

## Materials and Methods

### Human subjects

The study was approved by the Institutional Review Board of the University of Southern California. It involved anonymous organ donors and was certified as exempt: 45 CFR 46.101 (b) (4).

### Source of testes

Testes were obtained from the National Disease Research Interchange (NDRI, Philadelphia, PA). No donors were accepted if they had been treated with drugs known to interfere with normal spermatogenesis. All samples were frozen within 10–12 h after death.

### Testis dissection

Details of the dissections, DNA isolation and quantitation of the amount of DNA in each testis piece have been published [26], [27].

### MEN2B mutation frequency assay

We modified a highly specific amplification assay that uses dideoxy-terminated PCR primers in a reaction buffer with added pyrophosphate (pyrophosphorolysis-activated PCR, or PAP [28]). In general our assay is almost identical to that used in our earlier work [26], [27], [61]. Each MEN2B amplification reaction contained 20 mM HEPES (pH 7.0), 30 mM KCl, 50 µM Na_4_PPi, 2 mM MgCl_2_, 80 µM of each dNTP and 160–320 nM of each primer. The MEN2B specific primer sequences were 5′TGCGTGGTGTAGATATGATCAAAAAGGGATTCAATTGCCG_dd_3′ (Biosearch) and 5′ TCCATCTTCTCTTTAGGGTCGGATTCCAGTTAAATGGAC_dd_ 3′ (IDT). Each reaction also included 2 µM Rox, 0.2 X Syber Green I, 0.04 unit/uL TMA31FS DNA polymerase (Roche Molecular Systems), and DNA containing 25,000 genomes from the testis piece. [Research samples of Tma31FS DNA polymerase may be obtained from Dr. Thomas W. Myers, thomas.myers@roche.com, Director, Program in Core Research, Roche Molecular Systems, Inc., 4300 Hacienda Drive, Pleasanton, CA 94588]. PCR was carried out in 384 well plates using either a Roche LightCycler 480 or a Applied Biosystems 7900. The cycling conditions (Roche LightCycler 480) were: initial denaturation 1 min, 94°C and 130 cycles of 6 s, 94°C and 1 min, 73°C. Initial denaturation using the Applied Biosystems 7900 was 1 min, 94°C followed by 130 cycles of 6 s, 94°C and 1 min 15 s, 74.4°C. In this paper the false positive rate in the MEN2B assay was 4.7×10^−7^ based on an analysis of 269 million wild type genomes.

### Mutation counting strategy

We initially estimated the MEN2B mutation frequency using ten reactions (25,000 genomes per reaction) from every testis piece. If less than 5/10 reactions were positive we took that number as an estimate of the mutation frequency (after Poisson correction). If 5 or more reactions were positive we repeated the experiment using diluted samples of the piece until fewer than 50% were positive. The presence of a single mutant molecule in any reaction was detected by examining the kinetics of fluorescence increase as a function of cycle number using quantitative PCR and evaluation of the PCR product melting profile. Sample PAP data has already been published as a supporting figure in an earlier publication [27].

In every experiment 20 negative controls each contained 25,000 human blood genomes from unaffected individuals (Promega). Twenty positive controls each contained 25,000 control blood genomes and an average of 0.5 or 1 genome of MEN2B DNA with the c.2943T>C mutation (kindly provided by Dr. Robert Hofstra). The estimate of the total testis mutation frequency (mutations per million genomes) was the average of the frequencies of the pieces weighted by the number of genomes in those pieces.

### Quantitative modeling and testing

The computer code and instructions to simulate all of the models can be found in Protocol S1.

## Supporting Information

Dataset S1 MEN2B mutation frequency estimates for all testes' pieces.
(XLS)

Dataset S2 Apert syndrome mutation frequency estimates for all testes' pieces.
(XLS)

Figure S1 Distribution of the Apert syndrome c.755C>G mutation in 15 testes. Each testis is cut into 6 slices, and each slice is further dissected into 32 approximately equal pieces. The mutation frequency per million genomes (pmg) in each piece is represented by the color code in the lower left-hand corner of the figure. Above each testis is its identification number and the age of the donor. The testes are organized by age: the left-hand column is the youngest age group (19 to 23 years), the middle column is the middle-aged group (36 to 68 years), and the right-hand column is the oldest age group (75 to 80 years). 14 of these testes were shown in Figure 1 for the MEN2B mutation; for testis 63205 (age 21), we only measured the Apert mutation frequency so it is in this figure but not Figure 1. The figures for six testes (59056, 60832, 59089, 854-2, 374-1, and 374-2) have been published previously [26], [27].
(EPS)

Protocol S1 Computer code and brief instructions.
(DOC)

Table S1 Apert syndrome c.755C>G mutation: selection model incorporating cell death model parameters and simulation results for donors over age 40.
(DOC)

Text S1 The unexpectedly high incidence of MEN2B.
(DOC)

Text S2 The uniformity assumption.
(DOC)

Text S3 Details of the selection model incorporating cell death.
(DOC)
