# Supplementary material for: Positive Selection for New Disease Mutations in the Human Germline: Evidence from the Heritable Cancer Syndrome Multiple Endocrine Neoplasia Type 2B
Source: PLoS Genet. 2012 Feb 16;8(2):e1002420. doi: 10.1371/journal.pgen.1002420 (PMC3280958; doi:10.1371/journal.pgen.1002420)
Supplement: Protocol S1 — Computer code and brief instructions. (DOC) [file pgen.1002420.s004.doc]

**PROTOCOL S1. COMPUTER CODE AND BRIEF INSTRUCTIONS**

The files allmod.h and allmod.cpp contain the computer code to simulate the models discussed in the manuscript. The code is written in the C++ language and uses mpi parallelization. Once the code is compiled, the command line inputs are (in order): random number seed, age of the individual (in years), mutation rate per cell division, selection parameter p, cell death parameter delta, number of simulations, model number (2 means selection model incorporating cell death, 1 means symmetric variant to the hotspot model, and 0 means the original hot spot model or the selection model not incorporating cell death), and symmetric parameter q. For each simulation the outputs are (in order): age of the individual (in years), model number, selection parameter p, mutation rate per cell division, symmetric parameter q, cell death parameter delta, average testis mutation frequency, fraction of testis pieces required to contain 95% of mutations, maximum piece mutation frequency, minimum piece mutation frequency, and fraction of testis pieces with mutation frequencies less than 50 mutants per million genomes.

Detail: near the top of the allmod.h file one can change the constants PARALLEL for the number of computer processors used and DECREASEAGE (1 means incorporate cell death, 0 means do not incorporate cell death).

**ALL.H**

#include <mpi.h>

#include <iostream>

#include <fstream>

#include <iomanip>

#include <cmath>

#include <stdlib.h>

using namespace std;

#ifndef ALLMOD_H

#define ALLMOD_H

#define PARALLEL 64 // this is parallel code, so the number of processes

#define DECREASEAGE 1 // 1 incorporates cell death, 0 does not

void myseed(int bseed) {

MPI_Barrier(MPI_COMM_WORLD);

int myid,i,seed;

MPI_Status status;

MPI_Comm_rank(MPI_COMM_WORLD, &myid);

if (myid == 0) {

srand48(bseed);

for (i=1;i<PARALLEL;i++) {

seed = ((int)(ceil(drand48()*1000000.0)));

MPI_Send(&seed,1,MPI_INT,i,1,MPI_COMM_WORLD);

}

}

else {

for (i=1;i<PARALLEL;i++) {

if (myid == i) {

MPI_Recv(&seed,1,MPI_INT,0,1,MPI_COMM_WORLD,&status);

srand48(seed);

}

}

}

MPI_Barrier(MPI_COMM_WORLD);

}

double normal(double mean,double sd) {

double y1,y2,x;

int flag;

flag = 1;

while (flag) {

y1 = -log(drand48());

y2 = -log(drand48());

if (y2 >= (0.5*(y1-1.0)*(y1-1.0))) {

if (drand48() < 0.5) { x = y1; }

else { x = -y1; }

flag = 0;

}

}

x = mean + (x*sd);

return x;

}

int poisson(double x) {

double temp;

int flag, cnt;

temp = 0.0;

flag = 1;

cnt = 0;

while (flag) {

cnt += 1;

temp = temp + log(drand48());

if (temp < (-x)) {

flag = 0;

cnt -= 1;

}

}

return cnt;

}

double myrand(double num,double q) {

// POISSON. IF NUM*Q HIGH THEN NORMAL APPROXIMATION TO SPEED UP THE ALGORITHM

double dnum,temp;

if ((num > 0.0) && (q > 0.0)) {

dnum = num*q;

if (dnum < 100.0) {

temp = ((double)(poisson(dnum)));

}

else {

temp = round(normal(dnum,(sqrt(dnum))));

}

if (temp < 0.0) { temp = 0.0; }

if (temp > num) { temp = num; }

}

else { temp = 0.0; }

return temp;

}

double grow(double sta,int stime,int etime,double p,double symm,double overdeath,int model) {

// OUTPUT NUMBER OF MUTANTS IN THE CLUSTER

// INPUT INITIAL NUMBER MUTANTS (1 FOR ADULT PHASE, MORE FOR GROWTH PHASE)

// stime and etime in cell generations not years

int i;

double x,y,z,d;

x = sta;

if (model == 0) { // ORIGINAL HOTSPOT/SELECTION MODEL

if (p > 0.0) {

for (i=stime;i<etime;i++) {

x += myrand(x,p);

}

}

}

if (model == 1) { // SYMMETRIC VARIANT TO THE HOTSPOT MODEL

if (symm > 0.0) {

for (i=stime;i<etime;i++) {

y = 0.0;

for (i=0;i<x;i++) {

if (drand48() < (2.0*symm)) {

if (drand48() < symm) { y += 1.0; }

else { y -= 1.0; }

}

}

x += y;

if ( x <= 0.0) {

x = 0.0;

break;

}

}

}

}

if (model == 2) { // SELECTION MODEL INCORPORATING CELL DEATH

for (i=stime;i<etime;i++) {

d = 0.0; // CAN SPEED UP BY ONLY CHECKING EVERY ~10 YEARS

if (i >= ((35-13)*23)) { d = 0.00013; }

if (i >= ((45-13)*23)) { d = 0.00086; }

if (i >= ((55-13)*23)) { d = 0.00032; }

if (i >= ((65-13)*23)) { d = 0.00170*overdeath; }

if (i >= ((75-13)*23)) { d = 0.00150*overdeath; }

y = myrand(x,p);

z = myrand(x,d);

x = x + y - z;

if (x <= 0.0) {

x = 0.0;

break;

}

}

}

return x;

}

void space(double testis[6][8][4],double mclump,double mtot) {

// OUTPUT DISTRIBUTES MUTATION CLUSTER INTO 3-DIMENSIONAL TESTIS ARRAY

// INPUT 3-DIMENSIONAL TESTIS ARRAY,

// NUMBER OF MUTANTS IN CLUMP, TOTAL NUMBER OF CELLS IN TESTIS

double x,y,z,x1,x2,y1,y2,z1,z2,leng,dtemp;

int i,j,k;

x = 6.*drand48();

y = 8.*drand48();

z = 4.*drand48();

leng = pow( 192.*mclump/mtot, 0.333);

if (leng > 4) {

dtemp = round(mclump/192.);

for (i=0;i<6;i++) {

for (j=0;j<8;j++) {

for (k=0;k<4;k++) {

testis[i][j][k] += dtemp;

if (testis[i][j][k] > (mtot/192.)) {

testis[0][0][0] = -9;

}

}

}

}

}

else {

if (x-(0.5*leng) < 0) { x += (0.5*leng)-x; }

if (x+(0.5*leng) > 6) { x -= x+(0.5*leng)-6; }

if (y-(0.5*leng) < 0) { y += (0.5*leng)-y; }

if (y+(0.5*leng) > 8) { y -= y+(0.5*leng)-8; }

if (z-(0.5*leng) < 0) { z += (0.5*leng)-z; }

if (z+(0.5*leng) > 4) { z -= z+(0.5*leng)-4; }

for (i=0;i<6;i++) {

for (j=0;j<8;j++) {

for (k=0;k<4;k++) {

x1 = max( ((double)(i)), (x-(0.5*leng)) );

x2 = min( ((double)(i+1)),(x+(0.5*leng)) );

y1 = max( ((double)(j)), (y-(0.5*leng)) );

y2 = min( ((double)(j+1)),(y+(0.5*leng)) );

z1 = max( ((double)(k)), (z-(0.5*leng)) );

z2 = min( ((double)(k+1)),(z+(0.5*leng)) );

if ((x2 > x1) && (y2 > y1) && (z2 > z1)) {

dtemp = (x2-x1)*(y2-y1)*(z2-z1);

testis[i][j][k] += round( mclump*dtemp/pow(leng,3.) );

if (testis[i][j][k] > (mtot/192.) ) {

testis[0][0][0] = -9;

}

}

}

}

}

}

}

void simm(double p,double lambda,int yrs,double testis[6][8][4],int model,double symm,double overdeath) {

int growgens = 30;

double fp = 0.015;

int iii,jjj,cnt,divyr,adultgens;

adultgens = (yrs-13)*23;

int i,j,k,itime;

double mtot,extra,dtemp,muts,x,totmut,d;

totmut = 0.0;

mtot = round(pow(2,((double)(growgens))));

// FORMAT

extra = 0;

for (i=0;i<6;i++) {

for (j=0;j<8;j++) {

for (k=0;k<4;k++) {

testis[i][j][k] = 0.0;

}

}

}

// GROWTH PHASE

for (i=0;i<growgens;i++) {

dtemp = pow(2,((double)(i+1)));

muts = myrand(dtemp,lambda);

if (muts > 0) {

dtemp = pow(2,((double)(growgens-i-1)));

for (j=0;j<muts;j++) {

x = grow(dtemp,0,adultgens,p,symm,overdeath,model);

totmut += x;

extra += (x-dtemp);

space(testis,x,mtot);

if (testis[0][0][0] < 0.0) { break; }

}

//if (testis[0][0][0] < 0.0) { break; }

}

}

// ADULT PHASE

if (testis[0][0][0] >= 0.0) {

for (i=0;i<adultgens;i++) {

if (DECREASEAGE == 1) {

d = 0.0; // CAN SPEED UP BY ONLY CHECKING EVERY ~10 YEARS

if (i >= ((35-13)*23)) { d = 0.00013; }

if (i >= ((45-13)*23)) { d = 0.00086; }

if (i >= ((55-13)*23)) { d = 0.00032; }

if (i >= ((65-13)*23)) { d = 0.00170; }

if (i >= ((75-13)*23)) { d = 0.00150; }

mtot -= myrand(mtot,d);

}

muts = myrand(mtot,lambda);

if (muts > 0) {

for (j=0;j<muts;j++) {

x = grow(1.0,i,adultgens,p,symm,overdeath,model);

totmut += x;

extra += x;

space(testis,x,mtot);

if (testis[0][0][0] < 0.0) { break; }

}

//if (testis[0][0][0] < 0.0) { break; }

}

}

}

// INFERENCE

if (testis[0][0][0] >= 0.0) {

//mtot += round(extra);

double dil,trufreq,pos,lambda,lothresh,hithresh;

int ii,jj,tot,flag;

tot = 10;

lothresh = 2.0;

hithresh = 8.0;

for (i=0;i<6;i++) {

for (j=0;j<8;j++) {

for (k=0;k<4;k++) {

trufreq = testis[i][j][k]/(mtot/192.0);

dil = 1.0;

flag = 1;

while (flag) {

lambda = 25000.0*trufreq/dil;

pos = 0.0;

for (ii=0;ii<tot;ii++) {

jj = poisson(lambda);

if (jj > 0) { pos += 1.0; }

else {

if (drand48() < fp) { pos += 1.0; }

}

}

if (pos >= hithresh) { dil = 2.0*dil; }

else {

if (pos <= lothresh) {

if (dil == 1.0) { flag = 0; }

else { dil = 0.75*dil; }

}

else { flag = 0; }

}

}

testis[i][j][k] = ( -40.0*dil*( log(1.0-(pos/((double)(tot)))) - log(1.0-fp) ) )/1000000.0;

if (testis[i][j][k] < 0.0) { testis[i][j][k] = 0.0; }

}

}

}

}

}

double getp95(double testis[6][8][4]) {

// OUTPUT THE PERCENTAGE OF TESTIS PIECES REQUIRED TO COMPRISE 95% OF THE MUTANTS

// INPUT 3-DIMENSIONAL TESTIS ARRAY

int i,j,k,cnt,ibig;

double big,tot,lin[192];

cnt = -1;

for (i=0;i<6;i++) {

for (j=0;j<8;j++) {

for (k=0;k<4;k++) {

cnt += 1;

lin[cnt] = testis[i][j][k];

tot += testis[i][j][k];

}

}

}

for (i=0;i<191;i++) {

big = lin[i];

for (j=(i+1);j<192;j++) {

if (lin[j] > big) {

big = lin[j];

ibig = j;

}

}

if (big > lin[i]) {

lin[ibig] = lin[i];

lin[i] = big;

}

}

// THIS IS A CHANGE FROM THE PUBLISHED CODE

cnt = -1;

big = 0.;

while ((big/tot) < 0.95) {

cnt += 1; if (cnt > 191) { cnt = 191; break; }

big += lin[cnt];

}

return ((double)(cnt+1))/192;

}

void stat4(double testis[6][8][4],double xstat[]) {

int i,j,k;

if (testis[0][0][0] < 0.0) {

xstat[0] = -9;

xstat[1] = -9;

xstat[2] = -9;

xstat[3] = -9;

xstat[4] = -9;

}

else {

xstat[0] = 0.0;

xstat[2] = testis[0][0][0];

xstat[3] = testis[0][0][0];

xstat[4] = 0.0;

for (i=0;i<6;i++) {

for (j=0;j<8;j++) {

for (k=0;k<4;k++) {

xstat[0] += testis[i][j][k];

if (testis[i][j][k] > xstat[2]) {

xstat[2] = testis[i][j][k];

}

if (testis[i][j][k] < xstat[3]) {

xstat[3] = testis[i][j][k];

}

if ((testis[i][j][k]*1000000.) < 50.) {

xstat[4] += 1.0;

}

}

}

}

xstat[0] = (xstat[0]/192.)*1000000.; // FREQ

xstat[2] = xstat[2]*1000000.; // MAX

xstat[3] = xstat[3]*1000000.; // MIN

xstat[1] = getp95(testis);

xstat[4] = xstat[4]/192.0; // frac pieces <= 50 mutants per million molecules

}

}

void onesimm(double p,double lambda,int yrs,int model,double symm,double overdeath) {

double testis[6][8][4];

int i,j,k;

simm(p,lambda,yrs,testis,model,symm,overdeath);

for (i=0;i<6;i++) {

for (j=0;j<8;j++) {

for (k=0;k<4;k++) {

cout << testis[i][j][k] << endl;

}

}

}

}

void manysimm(double p,double lambda,int yrs,int model,double symm,double overdeath,int nreps) {

MPI_Barrier(MPI_COMM_WORLD);

int myid;

MPI_Status status;

MPI_Comm_rank(MPI_COMM_WORLD,&myid);

double testis[6][8][4],xstat[5];

int i,deli,flag,cont,half,top;

deli = -1;

flag = 1;

while (flag) {

if (myid > 0) {

simm(p,lambda,yrs,testis,model,symm,overdeath);

stat4(testis,xstat);

MPI_Send(xstat,5,MPI_DOUBLE,0,11,MPI_COMM_WORLD);

MPI_Recv(&flag,1,MPI_INT,0,12,MPI_COMM_WORLD,&status);

}

else {

if (PARALLEL > 1) {

MPI_Recv(xstat,5,MPI_DOUBLE,MPI_ANY_SOURCE,11,MPI_COMM_WORLD,&status);

}

else {

simm(p,lambda,yrs,testis,model,symm,overdeath);

stat4(testis,xstat);

}

deli += 1;

// OUTPUT

cout << yrs << " " << model << " " << p << " " << lambda << " " << symm << " " << overdeath << " " << xstat[0] << " " << xstat[1] << " " << xstat[2] << " " << xstat[3] << " " << xstat[4] << endl;

if ((PARALLEL > 1) && (deli <= (nreps-PARALLEL))) { cont = 1; }

else {

cont = 0;

if (deli == (nreps-1)) {

flag = 0;

}

}

if (PARALLEL > 1) {

MPI_Send(&cont,1,MPI_INT,status.MPI_SOURCE,12,MPI_COMM_WORLD);

}

}

}

MPI_Barrier(MPI_COMM_WORLD);

}

#endif

**ALL.CPP**

#include "allmod.h"

main(int argc, char *argv[]) {

MPI_Init(&argc,&argv);

int myid;

MPI_Status status;

MPI_Comm_rank(MPI_COMM_WORLD,&myid);

int seed;

seed = atoi(argv[1]); // random number seed

myseed(seed);

int targetage,nreps;

double lambda,p,overdeath;

targetage = atoi(argv[2]); // age of individual (in years)

lambda = atof(argv[3]); // mutation rate per cell division

p = atof(argv[4]); // selection parameter

overdeath = atof(argv[5]); // delta parameter

nreps = atoi(argv[6]); // number simulations

double symm;

int i,model;

model = atoi(argv[7]); // 2 selection model incorporating cell death, 1 symmetric variant to the hotspot model, 0 original hotspot/selection model

symm = atof(argv[8]); // symmetric parameter q

manysimm(p,lambda,targetage,model,symm,overdeath,nreps); // simulates the model following these parameters

MPI_Finalize();

return 1;

}
