# Supplementary material for: Positive Selection for New Disease Mutations in the Human Germline: Evidence from the Heritable Cancer Syndrome Multiple Endocrine Neoplasia Type 2B
Source: PLoS Genet. 2012 Feb 16;8(2):e1002420. doi: 10.1371/journal.pgen.1002420 (PMC3280958; doi:10.1371/journal.pgen.1002420)
Supplement: Table S1 — Apert syndrome c.755C>G mutation: selection model incorporating cell death model parameters and simulation results for donors over age 40. (DOC) [file pgen.1002420.s005.doc]

**Table S1. Apert syndrome c.755C>G mutation: selection model incorporating cell death model parameters and simulation results for donors over age 40.**

|  |  | Model parameters | | Mx/Av^a^ | | F<50^b^ (%) | |
| --- | --- | --- | --- | --- | --- | --- | --- |
| Testis | Age | Mutation rate per cell division | Selection parameter | Data | Simulated 95% range | Data | Simulated 95% range |
| 59089 | 45 | 5.0 × 10^-11^ | 0.015 | 50 | 33 - 115 | 93 | 86 - 93 |
| 854-2 | 54 | 2.8 × 10^-11^ | 0.014 | 69 | 36 - 140 | 93 | 86 - 94 |
| 374-1 | 62 | 2.8 × 10^-11^ | 0.011 | 71 | 33 - 128 | 88 | 86 - 94 |
| 374-2 | 62 | 1.5 × 10^-11^ | 0.010 | 104 | 46 - 154 | 97 | 93 - 97 |
| 60891 | 68 | 2.6 × 10^-11^ | 0.012 | 52 | 33 - 119 | 95 | 85 - 95 |
| 64302 | 75 | 2.8 × 10^-11^ | 0.012 | 41 | 37 - 143 | 96 | 89 - 96 |
| 60954 | 76 | 5.6 × 10^-11^ | 0.010 | 82 | 29 - 92 | 97 | 90 - 97 |
| 60955 | 76 | 1.0 × 10^-11^ | 0.013 | 139 | 53 - 187 | 95 | 94 - 99 |
| 57650 | 80 | 3.6 × 10^-11^ | 0.013 | 82 | 32 - 114 | 88 | 85 - 93 |
| 60507 | 80 | 1.6 × 10^-11^ | 0.015 | 49 | 44 - 171 | 97 | 91 - 97 |

^a^Mx/Av ratio maximum mutation frequency piece to testis average mutation frequency

^b^F<50 fraction pieces with mutation frequency less than 50 mutants per million genomes
