# Supplementary material for: Positive Selection for New Disease Mutations in the Human Germline: Evidence from the Heritable Cancer Syndrome Multiple Endocrine Neoplasia Type 2B
Source: PLoS Genet. 2012 Feb 16;8(2):e1002420. doi: 10.1371/journal.pgen.1002420 (PMC3280958; doi:10.1371/journal.pgen.1002420)
Supplement: Text S1 — The unexpectedly high incidence of MEN2B. (DOC) [file pgen.1002420.s006.doc]

**Supporting Information Text S1**

**The unexpectedly high incidence of MEN2B**

New MEN2B mutations occur at an unexpectedly high frequency. The estimated prevalence of MEN2 cases is 2.9 x 10^-5^ (cited in [2]). Because MEN2B makes up at least 5-10% of all MEN2 cases the expected prevalence of MEN2B is 1.4-2.8 x 10^-6^. Since at least 50% of all MEN2B cases are sporadic [4,6] we estimate the frequency of new MEN2B c.2943T>C mutations at 0.7-1.4 x 10^-6^. Evolutionary sequence comparisons [10-15] and direct disease incidence data [16,17] on new mutations causing a variety of different genetic diseases suggest that T>C transitions arise at a frequency between 10^-8^ and 10^-9^. Assuming the average between these values, the frequency of new MEN2B c.2943T>C mutations is 140-280 times greater than expected which is especially surprising given that the site of this transition mutation is not at a CpG dinucleotide.
