# Supplementary material for: Positive Selection for New Disease Mutations in the Human Germline: Evidence from the Heritable Cancer Syndrome Multiple Endocrine Neoplasia Type 2B
Source: PLoS Genet. 2012 Feb 16;8(2):e1002420. doi: 10.1371/journal.pgen.1002420 (PMC3280958; doi:10.1371/journal.pgen.1002420)
Supplement: Text S2 — The uniformity assumption. (DOC) [file pgen.1002420.s007.doc]

**Supporting Information Text S2**

**The uniformity assumption**

Our approach is based on the assumption that new mutations should be uniformly distributed throughout the testis as are the human self-renewing Ap spermatogonial stem cells (SrAp). In the mouse it has been shown using microscopic analysis of seminiferous tubule cross-sections that mouse spermatogonial stem cells (SSC) appear non-uniformly distributed at the level of resolution (~1.0 x 10^-4^ mm^3^) of individual seminiferous tubules [1,2]. However, on the much larger scale of the human pieces we studied (~90 mm^3^) the SrAp are distributed uniformly throughout each piece and the whole testis. Finally, when we considered all the mutation distribution data (MEN2B and Apert) in all the testes together we found there is no region of the testis that is more or less likely to host a mutation cluster (calculations not shown). This further supports the “uniformity” assumption of SrAp cells in testes that forms the basis of our method.

1. Chiarini-Garcia H, Hornick JR, Griswold MD, Russell LD (2001) Distribution of type A spermatogonia in the mouse is not random. Biology of Reproduction 65: 1179-1185.

2. Yoshida S, Sukeno M, Nabeshima Y (2007) A vasculature-associated niche for undifferentiated spermatogonia in the mouse testis. Science 317: 1722-1726
