# Supplementary material for: Positive Selection for New Disease Mutations in the Human Germline: Evidence from the Heritable Cancer Syndrome Multiple Endocrine Neoplasia Type 2B
Source: PLoS Genet. 2012 Feb 16;8(2):e1002420. doi: 10.1371/journal.pgen.1002420 (PMC3280958; doi:10.1371/journal.pgen.1002420)
Supplement: Text S3 — Details of the selection model incorporating cell death. (DOC) [file pgen.1002420.s008.doc]

**Supporting Information Text S3**

**Details of the selection model incorporating cell death**

The selection model has two parameters: the mutation rate per cell division and the selection parameter p. The selection model incorporating cell death adds a third model parameter δ. Before age 65, we set the age-dependent rate of SrAp death per cell division to match the observed decrease in the number of SrAp cells [37]: before age 35 this rate is zero, between the ages of 35 and 45 this rate is 0.00013, between the ages of 45 and 55 this rate is 0.00086, between the ages of 55 and 65 this rate is 0.00032. The replacement of SrAp cells with transformed Ad cells makes the decrease in the number of SrAp cells just a lower bound for the amount of SrAp death. After age 65, we set the rate of SrAp death per cell division to be the rate needed to match the observed decrease in the number of SrAp cells multiplied by the new model parameter δ: from the ages of 65 to 75 this rate is 0.00170×δ, after age 75 this rate is 0.00150×δ. To balance this additional death, we then introduce new non-mutated SrAp cells, representing the activated Ad cells, such that the number of SrAp cells in the simulations corresponds to the experimental measurements [37]. After some computational experimentation, we set δ equal to 15 for all testes (and both the MEN2B and Apert syndrome mutations).

In order to address those testes without substantial mutation clusters we made two definitions. Considering the distribution of statistics in Tables 1 and 3, we defined a testis as being “typical of a young donor” if the Av is less than 20 pmg and the Mx is less than 100 pmg. Likewise, a testis is “typical of a middle-aged donor” if the Av is greater than 50 and the Mx/Av ratio is greater than 30. These definitions give more separation between the two types than our earlier definition of “substantial mutation clusters”. For the following simulations for MEN2B and older individuals (ages 75 and 80), we set the mutation rate per cell division equal to the median value in Table 4 (4.65×10^-11^). When we set the selection parameter equal to 0.008 (on the low end of the range in Table 4), 64% of the simulations of a 75 year old and 99% of the simulations of an 80 year old were typical of a young donor. When we increased the selection parameter to 0.0115 (the median value in Table 4), 94% of the simulations of a 75 year old and 59% of the simulations of an 80 year old were typical of a middle-aged donor. Thus a relatively slight variation in the selection parameter between individuals can explain the heterogeneity observed in the oldest age group in Table 1. As for the Apert syndrome mutation, SI Table 1 shows that the selection model incorporating cell death is consistent with those testes over age 40 (as before, for each testis the mutation rate per cell division and the selection parameter were varied to try to match both the Av and the Mx, and we only considered those simulations such that the simulated Av was within 5% of the observed Av). When we set the mutation rate per cell division equal to 2.80×10^-11^ and increased the selection parameter to 0.0125 (the median values for Apert in SI Table 1), 98% of simulations of a 75 year old and 89% of simulations of an 80 year old were typical of a middle-aged donor (using the same definitions of middle-aged donor we used for MEN2B) in agreement with Table 3. The inferred selection parameters are slightly higher for Apert than MEN2B (SI Table 1 and Table 4), as are the Av and Mx statistics (Table 3 and Table 1), and this increase can explain the difference in the older age groups between these two mutations. Finally for MEN2B and the youngest donors, even when we increased the mutation rate per cell division to 1.2×10^-10^ and the selection parameter to 0.02 (the two greatest values from Table 4), 97% of simulations of a 23 year old were typical of a young donor in agreement with Table 1. For these parameter values, the probability of a substantial mutation cluster developing in a 23 year old is very small due to the relatively low number of adult phase generations. Decreasing either of these model parameters or the age of the individual only increases the fraction of simulations typical of a young donor.
